# Electrochemical Oscillatory Baffled Reactors Fabricated with Additive Manufacturing for Efficient Continuous-Flow Oxidations

**DOI:** 10.1021/acssuschemeng.1c06799

**Published:** 2022-02-11

**Authors:** Elena Alvarez, Maria Romero-Fernandez, Diego Iglesias, Raul Martinez-Cuenca, Obinna Okafor, Astrid Delorme, Pedro Lozano, Ruth Goodridge, Francesca Paradisi, Darren A. Walsh, Victor Sans

**Affiliations:** † Departamento de Bioquimica, Biologia Molecular e Inmunologia, Facultad de Quimica, Universidad de Murcia, Campus Reg Excelencia Int Mare Nostrum, E-30100 Murcia, Spain; ‡ School of Chemistry, University of Nottingham, University Park, Nottingham NG7 2RD, United Kingdom; § Institute of Advanced Materials (INAM), Universitat Jaume I, Avda. Sos Baynat s/n, 12071 Castellon, Spain; ∥ Department of Mechanical Engineering and Construction, Universitat Jaume I, Av. Vicent Sos Baynat s/n, 12071 Castellon, Spain; ⊥ Faculty of Engineering, University of Nottingham, University Park, Nottingham NG7 2RD, United Kingdom; # The GSK Carbon Neutral Laboratory for Sustainable Chemistry, Jubilee Campus, University of Nottingham, Triumph Road, Nottingham NG7 2TU, United Kingdom; ∇ Department of Chemistry, Biochemistry and Pharmaceutical Sciences, University of Bern, Freiestrasse 3, 3012 Bern, Switzerland

**Keywords:** electrochemistry, oscillatory, flow, oxidation, continuous, 3D printing, additive manufacturing

## Abstract

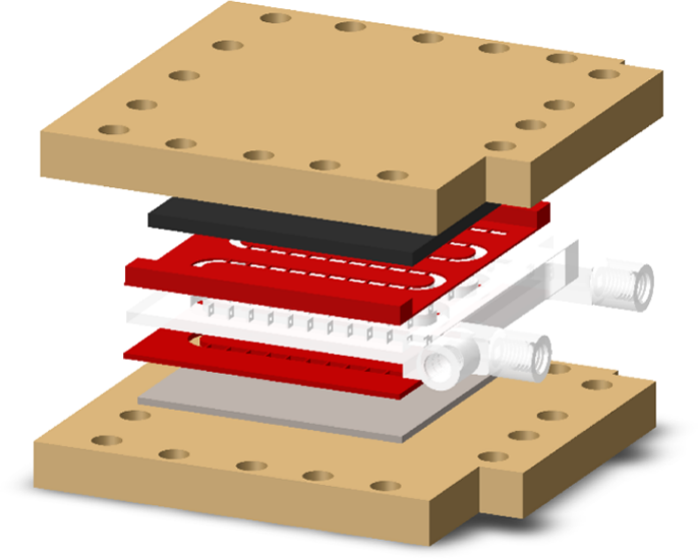

Electrochemical continuous-flow reactors offer a great opportunity for enhanced and sustainable chemical syntheses. Here, we present a novel application of electrochemical continuous-flow oscillatory baffled reactors (ECOBRs) that combines advanced mixing features with electrochemical transformations to enable efficient electrochemical oxidations under continuous flow at a millimeter distance between electrodes. Different additive manufacturing techniques have been employed to rapidly fabricate reactors. The electrochemical oxidation of NADH, a very sensitive substrate key for the regeneration of enzymes in biocatalytic transformations, has been employed as a benchmark reaction. The oscillatory conditions improved bulk mixing, facilitating the contact of reagents to electrodes. Under oscillatory conditions, the ECOBR demonstrated improved performance in the electrochemical oxidation of NADH, which is attributed to improved mass transfer associated with the oscillatory regime.

## Abstract 2

Continuous-flow electrochemical baffled reactors enhance mixing efficiency that improves mass transfer. The electrochemical oxidation of NADH has been demonstrated.

## Introduction

Continuous-flow synthetic methodologies are becoming a key technology for sustainable and efficient chemical manufacturing.^1^ Electrochemical flow reactors are gaining interest as alternative methodologies to perform chemical transformations in a clean and efficient fashion,^2−7^ especially when considering that energy can be obtained from renewable sources. This enables efficient transformations and reduces the amount of waste generated by avoiding the need for additives and auxiliary reagents to perform redox reactions. However, the performance of continuous-flow electroreactors can be affected by mass-transfer limitations of reagents and products to the electrodes if the reactor geometry is not carefully controlled.^2^ To address this, the electrodes are typically held apart by a few hundreds of microns, which improves mass transfer due to the small distances and it also minimizes Ohmic losses.^6^ These interelectrode distance requirements impose considerable limitations in the design of reactor architectures for continuous-flow electroreactors. Typically, flat parallel electrode configurations are chosen.^3^ Advanced reactor architectures could help to overcome these limitations by improving mixing, thus enabling efficient performance at a higher electrode distance. The higher reactor volume would, in turn, enable the integration of other enabling technologies for novel applications.^8^

In this regard, additive manufacturing (AM), commonly known as 3D printing, can offer new opportunities for the development of continuous-flow reactors,^9−11^ advanced reactor architectures,^12^ and other continuous-flow applications like crystallizers,^13,14^ calorimeters,^15^ or magnetic resonance probes.^16^ The digitalization of the fabrication process enables the coupling of advanced and lean design techniques, like computational fluid dynamics (CFD), to allow the generation of optimized reactor structures^17^ and the production of reactor geometries that are easily adaptable to commercial setups.^7^ Moreover, with the aid of CFD simulations, the shape of the reactor and the position of other elements like static mixers can be fine-tuned to optimize flow dynamics and consequently reactor performance before manufacturing.^18^

A key advantage of flow chemistry is the ability to finely control mixing, which can be done by adding elements without moving parts (passive),^19^ or with the use of an external energy source that produces changes in the flow resulting in better mixing.^20^ The use of AM to generate advanced structures that enhance mixing in electrochemical flow systems is gaining attention.^8,21,22^ Indeed, the design of simple structures to improve mixing in parallel plate continuous-flow electroreactors has been recently demonstrated, showcasing the potential of AM to improve the performance of electrochemical transformations under flow conditions.^23^ Continuous-flow oscillatory baffled reactors (COBRs) are a specific class of chemical reactors that combine both passive and active mixing: the coupling of periodic constrictions and a mechanically generated oscillatory flow enables the generation of a turbulent flow at low Reynolds (*Re*) numbers.^24,25^ The employment of additive manufacturing to generate miniaturized continuous oscillatory baffled reactors has been demonstrated previously by our group.^12^

Here, we report, for the first time, the design and fabrication of an electrochemical oscillatory baffled continuous-flow reactor (ECOBR), which combines enhanced mixing in electrochemical systems, allowing for efficient anodic oxidations. The improved mixing was demonstrated by CFD studies and preliminary work with the designed reactor to show the effect of oscillation in improving cell performance. Enhanced performance of the ECOBR can be comparable to a commercial parallel plate flow cell for electrochemical oxidations of NADH under continuous flow and opens the door to couple multiple enabling technologies, like electrochemical transformations, continuous flow, and other enabling technologies (e.g., biocatalysis).^26^

## Experimental Section

### Production of the Additively Manufactured Electrochemical Flow Reactor

#### Reactor

A baffled reactor was designed with CAD software (Creo Parametrics) and fabricated by laser sintering from nylon polyamide 12 (PA12) with an EOS P100 selective laser sintering (SLS) printer. Parts were built with a 21 W laser power (16 W contour), a 2500 mm/s scan speed, a 0.25 mm scan spacing, a 172.5 °C build temperature, with a removable platform at 150 °C.

#### Gaskets

Gaskets were initially printed in Formlabs Form 2 stereolithography (SLA) and Form 3 low force stereolithography (LFS) 3D printers using commercially available resins (Formlabs Flexible). Gaskets with the shape of the reactor and the baffles with a thickness of 0.5–1 mm were successfully fabricated. Alternatively, 0.5 mm thick gaskets were fabricated using an Ultimaker S5 fused filament printer, employing a red thermoplastic polyurethane (TPU95) filament purchased from Ultimaker.

#### Outer Casing

Parts were modeled using 3D design software and fabricated using a Formlabs 3 low force stereolithography (LFS) printer loaded with Formlabs Clear resin.

### Proof-of-Concept of the Additively Manufactured Electrochemical Flow Reactor

Proof-of-concept experiments were carried out using the AM electroreactor equipped with 6 × 6 cm^2^ electrodes (working electrode (WE): glassy carbon; counter electrode (CE): stainless steel 316). Cell performance was monitored using potentiometry with a two-electrode system on a μStat400 potentiostat (Metrohm Dropsens) using the cables supplied by the manufacturer, at a flow rate of 0.1 mL min^–1^ and a 15 μA cm^–2^ current, using 0.1 M aqueous 2-amino-2-(hydroxymethyl)propane-1,3-diol hydrochloride (Tris–HCl) as an electrolyte, whose pH was adjusted to ca. 6.7 with sodium hydroxide. Oscillatory flow regimes were generated by a C3000 programmable pump (Tricontinent) equipped with a 5 mL syringe.

### Electrochemical Oxidation of NADH

Cyclic voltammetry (CV) for evaluating the electrochemical oxidation of NADH was recorded using an Autolab PGSTAT302 (EcoChemie) with a three-electrode configuration (WE: glassy Carbon; RE: silver; CE: platinum). The concentration of Tris–HCl was 80 and 100 mM and NADH was 15 mM. The pH was varied at 7 and 8.8.

An Ammonite 8 spiral cell (100 × 0.2 × 0.5 cm^3^) equipped with 8.5 cm electrodes (WE: carbon/PVDF; CE: stainless steel) was used as a control experiment for the electrochemical oxidation of an NADH enzymatic cofactor. The AM electroreactor was equipped with square 6 × 6 cm^2^ electrodes (WE: glassy carbon; CE: Stainless steel 316), and the oscillation was controlled using a programmable Tricontinent C3000 pump equipped with a 5 mL syringe. Experiments for both reactors were performed using an aqueous solution of 1 mM NADH in Tris–HCl (10 mM, pH = 7), and the electrical current was controlled using an Autolab PGSTAT302 (EcoChemie). The current density (A cm^–2^) and normalized reactor productivity (mol_NAD+_·mol_NADH_^–1^·min^–1^·cm^–2^) were calculated as follows
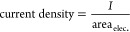
1
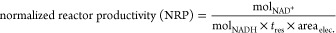
2
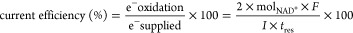
3where *I* is the intensity of current set with the potentiostat; area_elec_ is the geometric area of the electrode in contact with the reaction media; mol_NAD^+^_ and mol_NADH_ are the mols of the product and the reagent, respectively; *t*_res_ is the residence time; and *F* is the Faraday constant (96 485 C mol^–1^).

### Quantification of the NAD^+^ Concentration

In a typical experiment, an NADH solution in Tris–HCl was pumped through the reactor to fully load it and to reach the steady-state conditions. The residence time was calculated as the ratio between the volumetric flow rate and the total volume of the reactor. The NADH solution was pumped for the equivalent of two bed volumes, i.e., twice the residence time. Then, it was assumed that the steady state had been reached. The cell was maintained at constant current through this transient period and continued during the steady-state experiments. Applied currents were chosen using the Faraday equation (eq S1) applied to the reaction conditions (flow rate, concentration), with the aim of driving full conversion during the residence time. The NADH cofactor oxidation was quantified through its use in the enzymatic transformation of glucose into glucolactone employing a commercially available glucose dehydrogenase (GDH) enzyme. After the enzymatic reaction, the samples were analyzed in a UV–visible spectrophotometer at 340 nm and quantified through a calibration curve of NAD^+^ (Figures S1 and S2).

### CFD Simulations

The flow dynamics were resolved using the ANSYS-CFX 20 solver, based on the element-based finite volume method.^27^ Given the reduced Reynolds number (*Re*)^27,28^ of the flow, a transitional shear stress transport (SST) *k*–ω model turbulence model was used and the liquid was described as a Newtonian incompressible fluid. Two tracers were added to the flow, with a molecular diffusivity of 10^–8^ m^2^ s and a Schmidt number (ratio between turbulent eddy viscosity and eddy mass diffusivity)^27,29^ for turbulent dispersion of 0.9. The first tracer (inert tracer) entered through the inlet side and served to characterize the inlet flow mixing. The second tracer was generated as a mass flux, tracer mass (*m*_tracer_) per unit area and time, at the electrodes at a rate given by
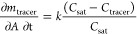
4In the simulations, *C*_sat_ = 0.1 kg m^–3^ and *k* = 1 mg mm^2^ s^–1^. *C*_tracer_ was the tracer volume concentration right next to the electrode and was calculated by the solver as it depends on the flow conditions.

Two transient simulations were performed to reproduce the flow advancement through the reactor. The inlet was modeled as an opening boundary condition so the fluid can enter and leave the flow domain. The velocity at this boundary was set as
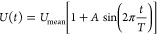
5with the values *U*_mean_ = 1 mm s^–1^ and *T* = 2 s. The amplitude was set to 0 for the steady-state case and *A* = 10 for the pulsating one.

## Results and Discussion

**Figure 1:**
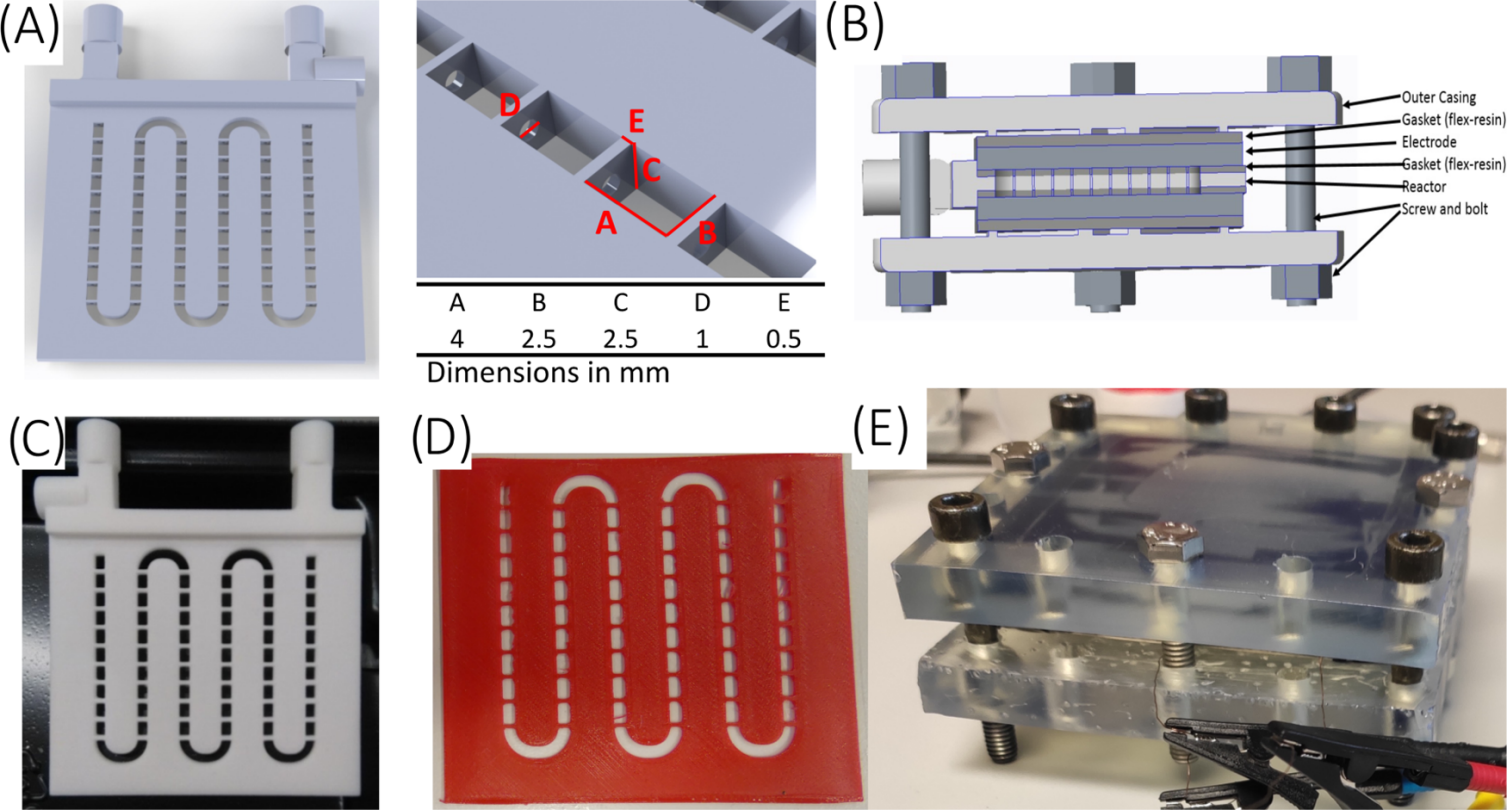
(A) General CAD design of the baffled section of the reactor (left), with a detailed view of the baffles (right) including the characteristic dimensions. (B) Schematic representation of ECOBR assembly. (C) Baffled reactor section fabricated in nylon employing selective laser sintering (SLS). (D) Gasket with tailored design matching baffles fabricated in TPU95 employing fused filament fabrication (FFF). (E) ECOBR assembly detail.

The initial design of the baffled reactor was based on the modification of a previously reported AM mCOBR reactor configuration.^12^ The channels were designed with a square section and open on both sides to accommodate the electrodes on the upper and lower walls of the reactor (Figure 1A). The reactor module is sandwiched with the electrodes on top and bottom, and the whole structure is completed with AM gaskets and support structures that are bolted together (Figure 1B). The reactor modules were printed by selective laser sintering (SLS) in polyamide 12 (PA12) (Figure 1C). Nylon is a robust, chemically resistant, and versatile material to generate structures that could be subsequently modified to add functionality.^30^ The structure is separated by gaskets fabricated with thermoplastic polyurethane (TPU95, Ultimaker) employing fused filament fabrication (FFF) technology (Figure 1D). The gaskets (0.5 mm thickness) were printed with geometries matching the baffles. Alternatively, the gaskets were fabricated with commercial flexible SLA resin (Flexible resin, Formlabs), but they were damaged upon utilization in the electrochemical setup (Figure S3). The design of the gaskets was modified to adapt to the size of the different electrodes. Stainless steel plates were cut to the size of the reactor chips and therefore required flat gaskets of the same size. The glassy carbon electrodes had narrower dimensions. Hence, the gaskets were modified to fill the gap between the electrode and the reactor module, thus facilitating the assembly of the ECOBR device, which was bolted together and tightened until no leak was observed by pumping distilled water through the reactor chamber at controlled flow rates. The pump flow rates were calibrated in the assembled setup to ensure that there were no leaks.

Proof-of-concept experiments were performed using the ECOBR assembled as previously described (Figure 1E), similarly to our previously reported method.^12^ A potentiostat was employed to measure the difference of electric potential under a constant current employing a two-electrode system, glassy carbon was used as a working electrode and stainless steel as a counter electrode (Figure S4). Oscillatory conditions were achieved by controlling the Tricontinent C3000 pump with in-house developed code using Labview (Figure S5). The parameters controlled by the user were frequency and amplitude of the oscillations, the latter actually controlling how much volume is displaced back and forth; it should be considered that due to the nature of the oscillatory movement, the net flow of the oscillatory action is zero.

**Figure 2:**
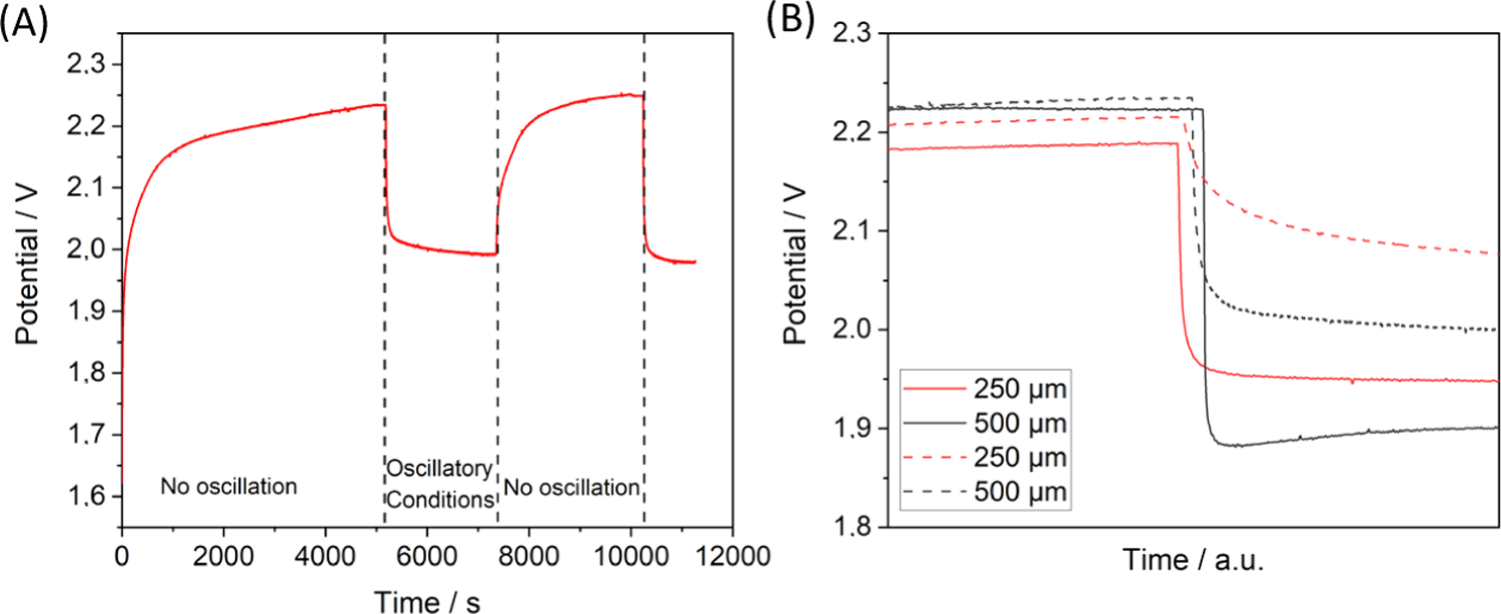
Chronoamperometry measurements of the ECOBR showing the effect of oscillatory conditions. (A) Activation and deactivation of the conditions. (B) Potential drop for different parameter combinations (amplitude: 250 μm for the red line and 500 μm for the black line. Frequency: 1 Hz for dashed lines and 5 Hz for solid lines).

The observed variations of the cell potential were due to the impact that oscillatory conditions have on the observed electrochemical process, presumably the electrolysis of the electrolyte (Tris–HCl). A flow of 0.1 mL min^–1^ was constantly pumped through the ECOBR while a current of 4.1 μA cm^–2^ was applied. Changes in potential were observed due to the differences in the operation of the reactor associated to different mixing conditions. We noted that when the cell was arranged with the cathode on top, the cell started behaving erratically and large bubbles were observed in the reactor outlet. Hence, the cathode was placed in the bottom to prevent gas evolution. Initially, no oscillatory regime was induced and the chamber was left to stabilize for 3 min. Afterward, a rapid increase in the potential was initially observed, followed by a stabilization period. The cell potential increased to above 2.2 V after 5000 s electrolysis (Figure 2A). However, once the syringe pump was activated and oscillatory conditions were initiated, the potential suddenly dropped and then slowly stabilized until it stabilized at below 2.0 V. If oscillation stopped, the potential grew again until it reached values similar to the original ones (Figure 2A).

The combined effect of oscillatory conditions and the baffled design of the reactor was expected to improve mass transport within the cell.^31^ This means that the transformation promoted by the ECOBR would be facilitated under oscillatory conditions.

The effect of the oscillation parameters on Δ*V*, i.e., the difference of potential observed before the oscillation started (*V*_0_) and the potential observed upon stabilization under oscillatory conditions, was examined. As shown in Table 1, an increase in frequency from 1 to 5 Hz is correlated with a decrease of the cell potentials (Table 1, entries 1 and 4). The effect of frequency achieved a maximum, evidenced by the lack of significant improvement at 10 Hz (Table 1, entry 7), in line with the behavior reported for COBRs in the literature.^12,25^ This has been explained as once a threshold frequency is trespassed, the effect of the oscillatory flow dominates over the net flow, the continuous reactor operates more likely to a batch reactor, and mixing is hindered.^32^

**Table 1:** Decrease in Cell Potential for Different Oscillation Parameter Combinations

**entry**	**amplitude (μm)**	**frequency (Hz)**	**Δ*VV***_**o**_^**–1**^ (**%)**
1	125	1	5.1
2	250	1	7.5
3	500	1	12.0
4	125	5	10.2
5	250	5	11.0
6	500	5	14.0
7	250	10	11.0

**Figure 3:**
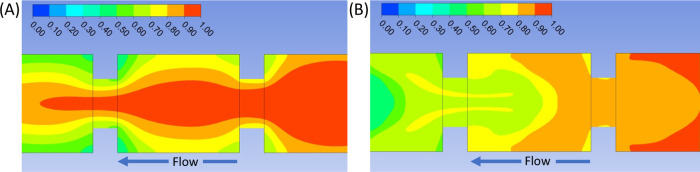
CFD simulation of the concentration of the inert tracer for (A) nonoscillating conditions and (B) oscillating conditions.

Flow dynamics were simulated in a virtual reactor with similar dimensions and operating conditions as the one used in the experimental part (Figure S7) to illustrate the enhanced mixing provided by the oscillating flow. The results demonstrate that the pulses of the oscillating conditions significantly enhance the mixing of the flow, thus improving the contact of the fluid with the walls of the reactor, which correspond to the electrodes. Figure 3 shows the concentration of the inert tracer across the reactor cross section. In the nonoscillating state (Figure 3A), the tracer remains close to the reactor axis (note the distribution in the central cell). The tracer distribution in the oscillating state is shown in Figure 3B. In this case, the tracer is distributed across the whole cell section, reaching the walls in a higher proportion, which should favor the electrochemical processes.

**Figure 4:**
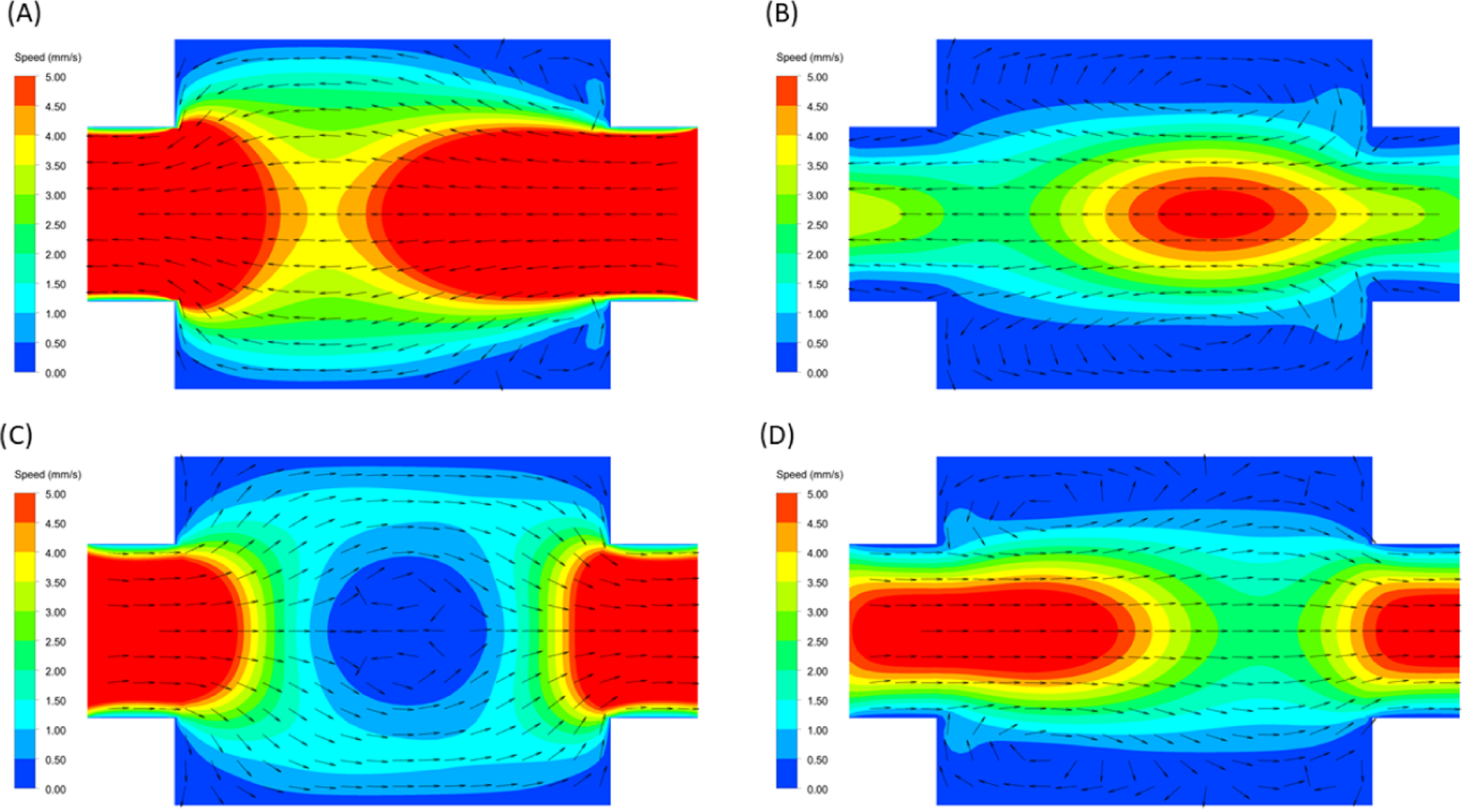
Results from CFD simulations for the velocity fields under oscillatory conditions. Color represents the flow speed and arrows indicate the flow direction (arrow size scales with velocity). Images from (A) to (D) correspond to different oscillation times inside the half cycle.

The improved mixing is due to the change of the flow paths, i.e., it is not related to flow induced turbulence. This can be noted in Figure 4, which shows the CFD calculated velocity fields at four different times within half an oscillating cycle. The flow in the nonoscillating case (Figure S8) is limited to the central region, flowing smoothly from the cell inlet toward the cell outlet. As the cell section is bigger than the tubular region, the flow slightly spreads but cannot efficiently reach the electrodes. Figure 4A–D shows the flow at different time steps under oscillatory conditions. These figures clearly show the formation of mixing vortexes (Figure 4B,D) and strong flow-reversal conditions (Figure 4C) near the electrodes. This flow field enhances the mixing compared to the nonoscillatory conditions (see Figure S7), characterized by low speeds near the electrodes and an even flow distribution.

Once the concept of the ECOBR was demonstrated, the next step was to evaluate its efficacy in a reaction process. The use of biocatalysts in industrial environments has grown since they allow the use of environmentally friendlier conditions and offer great selectivity. However, many enzymes require cofactors in their catalytic pathways, and since the cost of cofactors is usually high, their regeneration is essential.^33^ The NADH/NAD^+^ is a cofactor redox couple that intervenes in numerous interesting reactions, such as those catalyzed by dehydrogenase enzymes.^34^ Furthermore, the electrooxidation of NADH to NAD^+^ has been thoroughly studied over the years^35−37^ and used in applications such as biosensors.^34^ For all of these reasons, the NADH oxidation was chosen to evaluate our proposed reactor.

**Figure 5:**
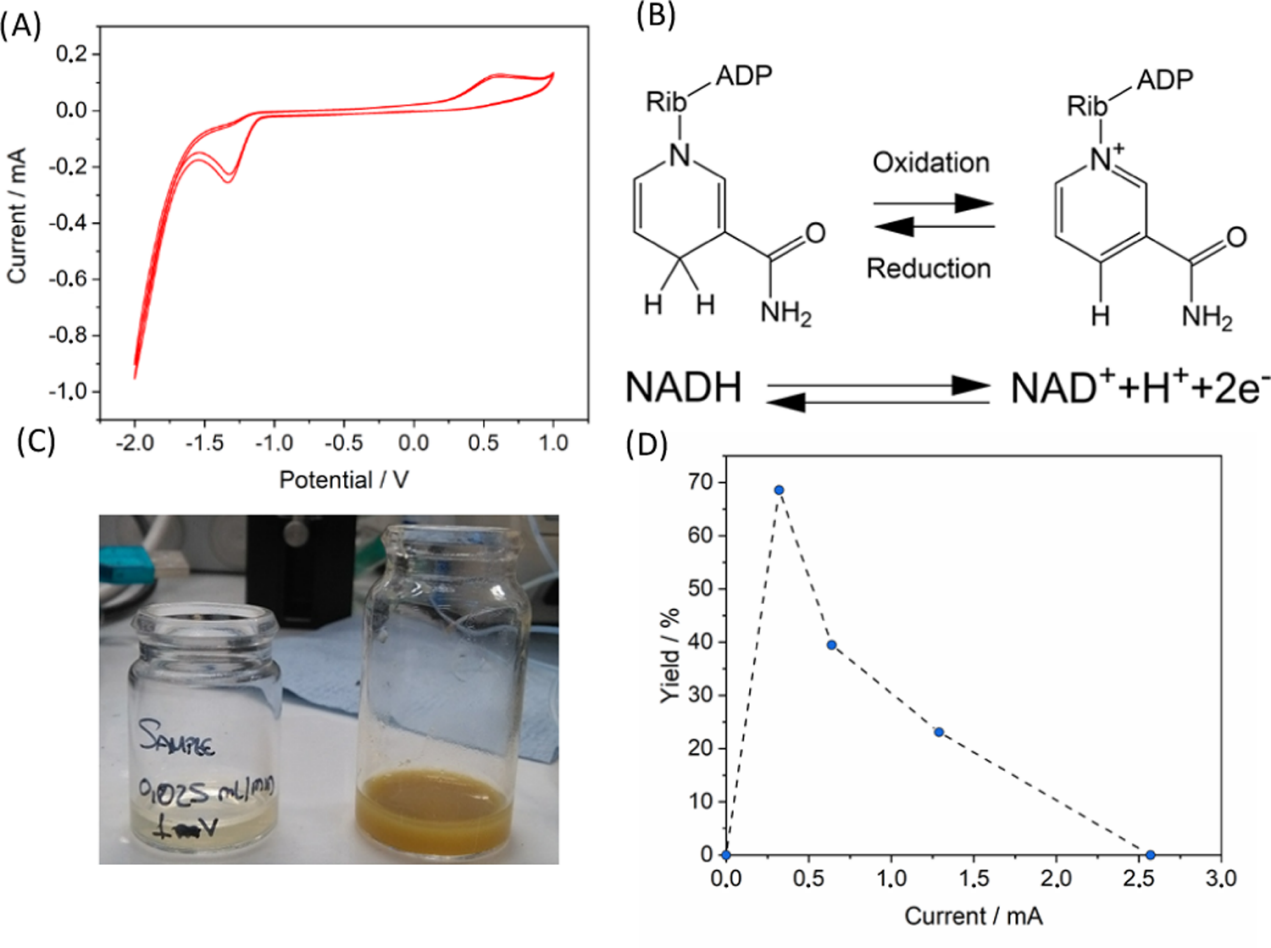
(A) Cyclic voltammetry of the oxidation of 15 mM NADH in 100 mM Tris–HCl (pH 7.0). WE glassy carbon, CE platinum, reference AgCl, and scan rate = 500 mV s^–1^. (B) Redox reaction of NADH. (C) Degraded samples after applying a high current. (D) Graph of yield vs current using the Ammonite 8 spiral reactor. Flow rate = 0.01 mL min^–1^, *t*_res_ = 100 min, room temperature, and [NADH] = 10 mM.

Before proceeding with the experiments using the reactor, cyclic voltammetry was performed using 15 mM NADH in Tris–HCl buffer to verify that the electrooxidation could take place in the reaction medium (Figure 5A, additional conditions and blank CVs are described in Figure S6). The voltammogram showed that NADH was oxidized to NAD^+^ (Figure 5A) at 0.6 V as previous works using glassy carbon have established,^38^ thus confirming that oxidation could be carried out in the ECOBR.

Initially, the testing under continuous-flow conditions was undertaken in a commercial Ammonite 8 electrochemical flow cell, which served as a benchmark of the oxidation reaction, which later was performed in the ECOBR. It is important to note that the reactor conditions are not strictly comparable in terms of performance and productivity due to the use of different current densities and electrode materials. The design of the Ammonite 8 reactor features a circular cell with a carved spiral pathway that the solution has to traverse while in contact with electrodes placed on the top and bottom walls of the reactor. The amount of NAD^+^ generated was quantified employing an enzymatic method (see the Supporting Information for more details and Figures S1 and S2); this also demonstrated that the electrochemically oxidized/regenerated cofactor is active in enzymatic reactions. Preliminary tests with a solution of NADH (10 mM) were performed with the cell potential fixed at 1 V, higher than required for the oxidation of NADH (Figure 5B), which caused the current to increase to 0.18 A and presumably caused the observed overoxidation (Figure 5C). The oxidation of NADH is a two-electron process, given a faradic efficiency of 100%; the amount of charge transferred would be equal to the amount of charge supplied of 2 F·mol^–1^ (*F* being 96 485 C). Hence, the current intensity was set to a value calculated with eq S1, providing the required amount of charge for a complete conversion during the residence time. For a residence time of 100 min and a molar flow rate of 0.01 μmol·min^–1^ (calculated as concentration × volumetric flow rate), eq S1 predicted a required current of 32 μA. Under these conditions, a stable regime was observed and the steady state was easily achieved. When a current above that calculated with eq S1 was applied, a negative effect on the yield of NADH was observed (Figure 5D and Table S1). This is presumably due to the overoxidation of the substrate at high current values.

The normalized reactor productivity (NRP) parameter (eq 2) was employed to compare the performance of the different experiments. Here, the NRP was calculated as the productivity of the reactor in terms of the molar flow rate of product (mol NAD^+^·min^–1^) per mol of reagent (NADH) and the electrode surface area along the reactor channels. Different values of current and flow rates were evaluated for the Ammonite 8 cell (Table 2, entries 1–3) and for the AM ECOBR without the oscillatory regime (Table 2, entries 4–7). In both reactors, the highest yield was achieved with the lowest flow rate, thus with longer residence time (Table 2, entry 1). The NRP of the Ammonite 8 reactor did not drop significantly as the flow rate was changed, which suggests that mass transfer in the cell was relatively efficient. It is important to keep in mind that the electrode distance is of ca. 0.2 mm. Next, the AM reactor was evaluated without using oscillatory conditions to find the optimum flow for this reactor. In this case, the electrodes are placed about 3 mm from each other, which is an order of magnitude higher than that in the commercial cell. This enables the integration of supplementary features, like the baffles, which can play additional roles for enhanced performance. Like for the commercial reactor, the highest yield was obtained with the lowest flow rate evaluated, corresponding to the highest residence time (Table 2, entry 4). The productivity correlates the moles of the product formed with the residence time of the reaction, representing the amount of the product generated per unit of time. Hence, the productivity does not necessarily correlate with the highest yield but with the highest reaction rate. Indeed, it was achieved with a residence time of 46 min (Table 2, entry 6). Therefore, these conditions were chosen for the oscillatory experiments.

**Table 2:** Results of the NADH Oxidation for Different Continuous-Flow Conditions

**entry**	**reactor**	**flow** (mL min^**–1**^)	***t***_**Res**_**(min)**	***I* (μA)**	**curr. dens.** (μA cm^**–2**^)	**Faradic efficiency (%)**	**normalized reactor productivity** × **10**^**4**^ (mol_**NAD+**_ **mol**_**NADH**_^**–1**^ **min**^**–1**^ **cm**^**–2**^)
1	Amm8	0.01	100	32	1.6	81	4.1
2	Amm8	0.02	50	64	3.2	39	3.9
3	Amm8	0.05	20	160	8.1	14	3.5
4	ECOBR	0.01	230	32	4.2	62	3.5
5	ECOBR	0.02	115	64	8.3	56	6.3
6	ECOBR	0.05	46	160	20.8	27	7.6
7	ECOBR	0.1	23	320	41.5	11	6.2

To elucidate the effect of the oscillations on the NRP, the current density was selected based on eq S1, which, as previously mentioned, calculates the stoichiometric amount of charge required for a full conversion of the electrolyzed reagents and it showed optimal performance with the Ammonite cell (see Figure 5D and Table S1, entry 2). Different combinations of amplitude and frequency were set using the flow rate and current previously selected, and their impact on the oxidation of NADH was evaluated in the ECOBR. The NRP values observed were higher than those under nonoscillating conditions for most of the combinations studied, except the one with the lowest amplitude (Table 3, entry 2). The best performance was achieved at an amplitude of 1 mm and a frequency of 0.5 Hz (Table 3, entry 3). The productivity observed was 2.2-fold higher than the same conditions without oscillating conditions.

**Table 3:** Results of NADH Oxidation for Different Conditions Using the AM Flow Reactor with Different Oscillation Conditions^a^

**Entry**	**λ (Hz)**	***A* (μm)**	**Faradic efficiency (%)**	**NRP** × **10**^**4**^ (**mol**_**NAD+**_ **mol**_**NADH**_^**–1**^ **min**^**–1**^ **cm**^**–2**^)
1	0	0	27	7.6
2	1	500	20	5.6
3	0.5	1000	60	16.9
4	1	1000	42	11.8
5	2	1000	31	8.7
6	1	1500	32	9.0

a Reaction conditions: flow rate = 0.05 mL min^–1^, *t*_res_ = 46 min, *I* = 160 μA, and current density = 20.8 μA cm^–2^.

**Figure 6:**
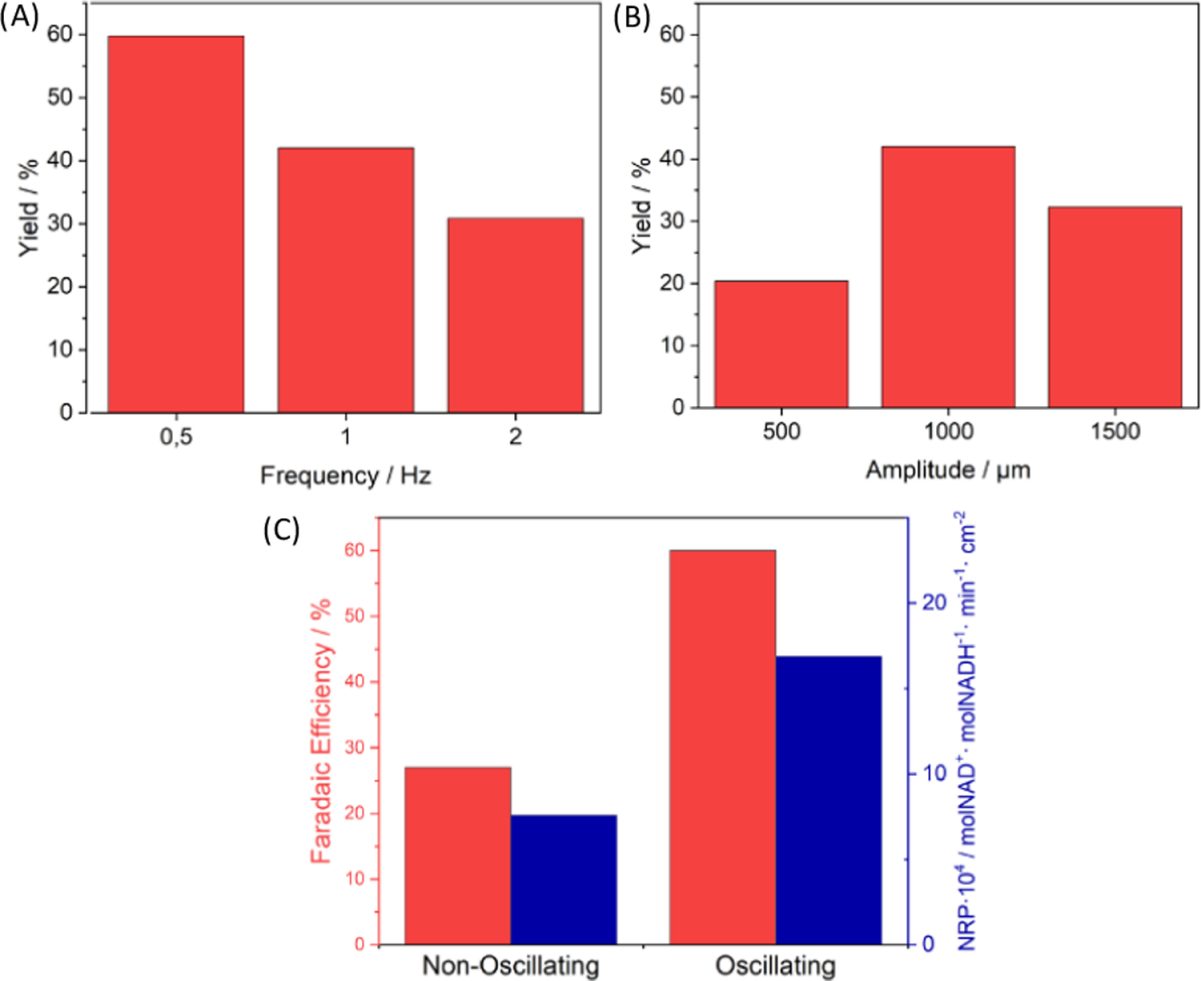
(A) Influence of the oscillation frequency on the yield of the AM ECOBR reactor (amplitude used: 1000 μm). (B) Influence of the oscillation amplitude on the yield (frequency used: 1 Hz). (C) Faradic efficiency and normalized reactor productivity (NRP) for the ECOBR under oscillating (*l* = 0.5 Hz, *A* = 1 mm) and nonoscillating conditions; both experiments were performed at following conditions: flow rate = 0.05 mL min^–1^, *t*_res_ = 46 min, I = 160 μA, and current density = 20.8 μA cm^–2^.

The oscillatory regime of the ECOBR demonstrated an effect on the yield of NAD^+^. Indeed, an increase in the frequency led to lower yields (Figure 6A), while the amplitude did not show a defined trend (Figure 6B). The generation of oscillations enhanced the performance of the ECOBR in terms of mols of NADH generated per unit of time and the surface area of the electrode.

Improving mass transport in the liquid phase can undoubtedly improve the performance of electrochemical flow reactors. In micro- and millireactors, laminar flow is predominant so there are different strategies to solve this problem; one of them is giving the reagents enough time to allow mixing by either lowering the flow or increasing the pathway.^39^ It is demonstrated that, as previously seen in the CFD simulations, the oscillations generated in the baffled reactor produce vortexes that improve the mixing, which could work as a different strategy to improve performance in electrochemical reactors (Figure 6C).

## Conclusions

In the present work, an electrochemical continuous-flow oscillatory baffled reactor was manufactured with different AM techniques. The design features a pathway segmented by numerous baffles that under a continuous oscillatory flow promote the generation of vortexes, which improve the mixing in the reactor. Different parameters of the amplitude and frequency of the oscillations, flow and current, were evaluated for the oxidation of NADH. The ECOBR under oscillatory flow conditions showed improved performance.

The simplicity of this reactor architecture paves the way for new applications that may arise thanks to the easy tuning enabled by AM. Additionally, the materials can be adapted to optimize each reaction system and the different components can even be functionalized to broaden their range of applications, thus envisioning the possibility of efficiently combining multiple processes in integrated devices; this offers novel windows of opportunity for sustainable chemical manufacturing.

## Supporting Information Available

Details on the assembly of the ECOBR, CVs of the oxidation of the buffer solution, Labview code and CFD conditions, experimental details about quantification of NAD+, and the use of different current conditions (PDF)

## References

[ref1] Gutmann B.; Cantillo D.; Kappe C. O. Continuous-Flow TechnologyA Tool for the Safe Manufacturing of Active Pharmaceutical Ingredients. Angew. Chem., Int. Ed. 2015, 54, 6688–6728. 10.1002/anie.201409318. 25989203

[ref2] Noël T.; Cao Y.; Laudadio G. The Fundamentals Behind the Use of Flow Reactors in Electrochemistry. Acc. Chem. Res. 2019, 52, 2858–2869. 10.1021/acs.accounts.9b00412. 31573791 PMC6796831

[ref3] Walsh F. C.; Ponce de León C. Progress in electrochemical flow reactors for laboratory and pilot scale processing. Electrochim. Acta 2018, 280, 121–148. 10.1016/j.electacta.2018.05.027.

[ref4] Maljuric S.; Jud W.; Kappe C. O.; Cantillo D. Translating batch electrochemistry to single-pass continuous flow conditions: an organic chemist’s guide. J. Flow Chem. 2020, 10, 181–190. 10.1007/s41981-019-00050-z.

[ref5] Kabeshov M. A.; Musio B.; Ley S. V. Continuous direct anodic flow oxidation of aromatic hydrocarbons to benzyl amides. React. Chem. Eng. 2017, 2, 822–825. 10.1039/C7RE00164A.

[ref6] Brown R. C. D. The Longer Route can be Better: Electrosynthesis in Extended Path Flow Cells. Chem. Rec. 2021, 21, 2472–2487. 10.1002/tcr.202100163. 34302434

[ref7] van Melis C. G. W.; Penny M. R.; Garcia A. D.; Petti A.; Dobbs A. P.; Hilton S. T.; Lam K. Supporting-Electrolyte-Free Electrochemical Methoxymethylation of Alcohols Using a 3D-Printed Electrosynthesis Continuous Flow Cell System. ChemElectroChem 2019, 6, 4144–4148. 10.1002/celc.201900815.

[ref8] Sans V. Emerging trends in flow chemistry enabled by 3D printing: Robust reactors, biocatalysis and electrochemistry. Curr. Opin. Green Sustainable Chem. 2020, 25, 100367 10.1016/j.cogsc.2020.100367.

[ref9] Kitson P. J.; Rosnes M. H.; Sans V.; Dragone V.; Cronin L. Configurable 3D-Printed millifluidic and microfluidic ’lab on a chip’ reactionware devices. Lab Chip 2012, 12, 3267–3271. 10.1039/c2lc40761b. 22875258

[ref10] Penny M. R.; Rao Z. X.; Peniche B. F.; Hilton S. T. Modular 3D Printed Compressed Air Driven Continuous-Flow Systems for Chemical Synthesis. Eur. J. Org. Chem. 2019, 2019, 3783–3787. 10.1002/ejoc.201900423.

[ref11] Rao Z. X.; Patel B.; Monaco A.; Cao Z. J.; Barniol-Xicota M.; Pichon E.; Ladlow M.; Hilton S. T. 3D-Printed Polypropylene Continuous-Flow Column Reactors: Exploration of Reactor Utility in SNAr Reactions and the Synthesis of Bicyclic and Tetracyclic Heterocycles. Eur. J. Org. Chem. 2017, 2017, 6499–6504. 10.1002/ejoc.201701111.

[ref12] Okafor O.; Weilhard A.; Fernandes J. A.; Karjalainen E.; Goodridge R.; Sans V. Advanced reactor engineering with 3D printing for the continuous-flow synthesis of silver nanoparticles. React. Chem. Eng. 2017, 2, 129–136. 10.1039/c6re00210b.

[ref13] Okafor O.; Robertson K.; Goodridge R.; Sans V. Continuous-flow crystallisation in 3D-printed compact devices. React. Chem. Eng. 2019, 4, 1682–1688. 10.1039/c9re00188c.

[ref14] Thomas K. M.; Kwon S.; Lakerveld R. Continuous Protein Crystallization in Mixed-Suspension Mixed-Product-Removal Crystallizers. Cryst. Growth Des. 2021, 21, 757–769. 10.1021/acs.cgd.0c00885.

[ref15] Maier M. C.; Leitner M.; Kappe C. O.; Gruber-Woelfler H. A modular 3D printed isothermal heat flow calorimeter for reaction calorimetry in continuous flow. React. Chem. Eng. 2020, 5, 1410–1420. 10.1039/d0re00122h.

[ref16] Xie J.; You X.; Huang Y.; Ni Z.; Wang X.; Li X.; Yang C.; Zhang D.; Chen H.; Sun H.; Chen Z. 3D-printed integrative probeheads for magnetic resonance. Nat. Commun. 2020, 11, 5793 10.1038/s41467-020-19711-y. 33188186 PMC7666178

[ref17] Bracconi M.; Ambrosetti M.; Okafor O.; Sans V.; Zhang X.; Ou X.; Da Fonte C. P.; Fan X.; Maestri M.; Groppi G.; Tronconi E. Investigation of pressure drop in 3D replicated open-cell foams: Coupling CFD with experimental data on additively manufactured foams. Chem. Eng. J. 2019, 377, 120123 10.1016/j.cej.2018.10.060.

[ref18] Bettermann S.; Kandelhard F.; Moritz H. U.; Pauer W. Digital and lean development method for 3D-printed reactors based on CAD modeling and CFD simulation. Chem. Eng. Res. Des. 2019, 152, 71–84. 10.1016/j.cherd.2019.09.024.

[ref19] Lee C.-Y.; Chang C.-L.; Wang Y.-N.; Fu L.-M. Microfluidic Mixing: A Review. Int. J. Mol. Sci. 2011, 12, 3263–3287. 10.3390/ijms12053263. 21686184 PMC3116190

[ref20] Hessel V.; Löwe H.; Schönfeld F. Micromixers—a review on passive and active mixing principles. Chem. Eng. Sci. 2005, 60, 2479–2501. 10.1016/j.ces.2004.11.033.

[ref21] Walsh F. C.; Arenas L. F.; Ponce de León C. Editors’ Choice—Critical Review—The Bipolar Trickle Tower Reactor: Concept, Development and Applications. J. Electrochem. Soc. 2021, 168, 023503 10.1149/1945-7111/abdd7a.

[ref22] Ambrosi A.; Shi R. R. S.; Webster R. D. 3D-printing for electrolytic processes and electrochemical flow systems. J. Mater. Chem. A 2020, 8, 21902–21929. 10.1039/D0TA07939A.

[ref23] Márquez-Montes R. A.; Collins-Martínez V. H.; Pérez-Reyes I.; Chávez-Flores D.; Graeve O. A.; Ramos-Sánchez V. H. Electrochemical Engineering Assessment of a Novel 3D-Printed Filter-Press Electrochemical Reactor for Multipurpose Laboratory Applications. ACS Sustainable Chem. Eng. 2020, 8, 3896–3905. 10.1021/acssuschemeng.9b07368.

[ref24] McDonough J.; Armett J.; Law R.; Harvey A. P. Coil-in-Coil Reactor: Augmenting Plug Flow Performance by Combining Different Geometric Features Using 3D Printing. Ind. Eng. Chem. Res. 2019, 58, 21363–21371. 10.1021/acs.iecr.9b04239.

[ref25] Reis N.; Vicente A. A.; Teixeira J. A.; Mackley M. R. Residence times and mixing of a novel continuous oscillatory flow screening reactor. Chem. Eng. Sci. 2004, 59, 4967–4974. 10.1016/j.ces.2004.09.013.

[ref26] Porcar R.; Sans V.; Ríos-Lombardía N.; Gotor-Fernández V.; Gotor V.; Burguete M. I.; García-Verdugo E.; Luis S. V. Stereoselective Chemoenzymatic Synthesis of Enantiopure 2-(1H-imidazol-yl)cycloalkanols under Continuous Flow Conditions. ACS Catal. 2012, 2, 1976–1983. 10.1021/cs300282w.

[ref27] ANSYS Inc. ANSYS Academic Research, Release 20.2, Help System, ANSYS CFX Reference Guide, 2020.

[ref28] Fogler S. H. Elements of Chemical Reaction Engineering, 5th ed.; Prentice Hall International: New Jersey, 2011.

[ref29] Tominaga Y.; Stathopoulos T. Turbulent Schmidt numbers for CFD analysis with various types of flowfield. Atmos. Environ. 2007, 41, 8091–8099. 10.1016/j.atmosenv.2007.06.054.

[ref30] Peris E.; Okafor O.; Kulcinskaja E.; Goodridge R.; Luis S. V.; Garcia-Verdugo E.; O’Reilly E.; Sans V. Tuneable 3D printed bioreactors for transaminations under continuous-flow. Green Chem. 2017, 19, 5345–5349. 10.1039/c7gc02421e.

[ref31] Lölsberg J.; Starck O.; Stiefel S.; Hereijgers J.; Breugelmans T.; Wessling M. 3D-Printed Electrodes with Improved Mass Transport Properties. ChemElectroChem 2017, 4, 3309–3313. 10.1002/celc.201700662.

[ref32] Avila M.; Fletcher D. F.; Poux M.; Xuereb C.; Aubin J. Mixing performance in continuous oscillatory baffled reactors. Chem. Eng. Sci. 2020, 219, 115600 10.1016/j.ces.2020.115600.

[ref33] Bommarius A. S.; Paye M. F. Stabilizing biocatalysts. Chem. Soc. Rev. 2013, 42, 6534–6565. 10.1039/C3CS60137D. 23807146

[ref34] Bartlett P. N.; Simon E.; Toh C. S. Modified electrodes for NADH oxidation and dehydrogenase-based biosensors. Bioelectrochemistry 2002, 56, 117–122. 10.1016/S1567-5394(02)00047-6. 12009456

[ref35] Moiroux J.; Elving P. J. Effects of adsorption, electrode material, and operational variables on the oxidation of dihydronicotinamide adenine dinucleotide at carbon electrodes. Anal. Chem. 1978, 50, 1056–1062. 10.1021/ac50030a015.

[ref36] Gorton L.; Domínguez E. Electrochemistry of NAD(P)+/NAD(P)H. In Encyclopedia of Electrochemistry; Wiley, 2007; pp 67–134.

[ref37] Immanuel S.; Sivasubramanian R. Electrochemical studies of NADH oxidation on chemically reduced graphene oxide nanosheets modified glassy carbon electrode. Mater. Chem. Phys. 2020, 249, 123015 10.1016/j.matchemphys.2020.123015.

[ref38] Wang S.; Yao Z.; Yang T.; Zhang Q.; Gao F. An Enzymatic Electrode Integrated with Alcohol Dehydrogenase and Chloranil in Liquid-Crystalline Cubic Phases on Carbon Nanotubes for Sensitive Amperometric Detection of NADH and Ethanol. J. Electrochem. Soc. 2019, 166, G116–G121. 10.1149/2.1341910jes.

[ref39] Maier M. C.; Lebl R.; Sulzer P.; Lechner J.; Mayr T.; Zadravec M.; Slama E.; Pfanner S.; Schmölzer C.; Pöchlauer P.; Kappe C. O.; Gruber-Woelfler H. Development of customized 3D printed stainless steel reactors with inline oxygen sensors for aerobic oxidation of Grignard reagents in continuous flow. React. Chem. Eng. 2019, 4, 393–401. 10.1039/c8re00278a.

