# Supplementary material for: Electrochemical Oscillatory Baffled Reactors Fabricated with Additive Manufacturing for Efficient Continuous-Flow Oxidations
Source: ACS Sustain Chem Eng. 2022 Feb 11;10(7):2388–96. doi: 10.1021/acssuschemeng.1c06799 (PMC8864614; doi:10.1021/acssuschemeng.1c06799)
Supplement: Supplementary file 1 — sc1c06799_si_001.pdf [file sc1c06799_si_001.pdf]

Electrochemical oscillatory baffled reactors fabricated with additive manufacturing for efficient continuous-flow oxidations

Elena Alvarez,[a] Maria Romero-Fernandez,[b] Diego Iglesias,[c] Raul Martinez-Cuenca,[d] Obinna Okafor,[e] Astrid Delorme,[f] Pedro Lozano,[a] Ruth Goodridge,[e] Francesca Paradisi,[g] Darren A. Walsh,[f] Victor Sans[c]*

a Universidad de Murcia, Facultad de Quimica, Dept Bioquimica, Biologia Molecular e Inmunologia, Campus Reg Excelencia Int Mare Nostrum, E-30100 Murcia, Spain.

b School of Chemistry, University of Nottingham. University Park, Nottingham, NG7 2RD, United Kingdom

c Institute of Advanced Materials (INAM), Universitat Jaume I, Avda. Sos Baynat s/n, 12071, Castellon, Spain. Corresponding author e-mail: sans@uji.es

d Department of Mechanical Engineering and Construction, Universitat Jaume I, Av. Vicent Sos Baynat, s/n, 12071 Castellon, Spain

e Faculty of Engineering, University of Nottingham. University Park, Nottingham, NG7 2RD (UK)

f The GSK Carbon Neutral Laboratory for Sustainable Chemistry. Jubilee Campus, University of Nottingham. Triumph Road, NG7 2TU Nottingham, United Kingdom

g Dept. of Chemistry, Biochemistry and Pharmaceutical Sciences, University of Bern, Freiestrasse 3, 3012 Bern, Switzerland

Table of contents

1 Gaskets S2
2 Programming of the syringe pump used for oscillation S3
3 CV of the oxidation of Tris-HCl S3
4 Geometry used for the CFD simulations S4
5 Study of higher currents in reaction....................S5
6 NAD+ quantification S6

1 Gaskets

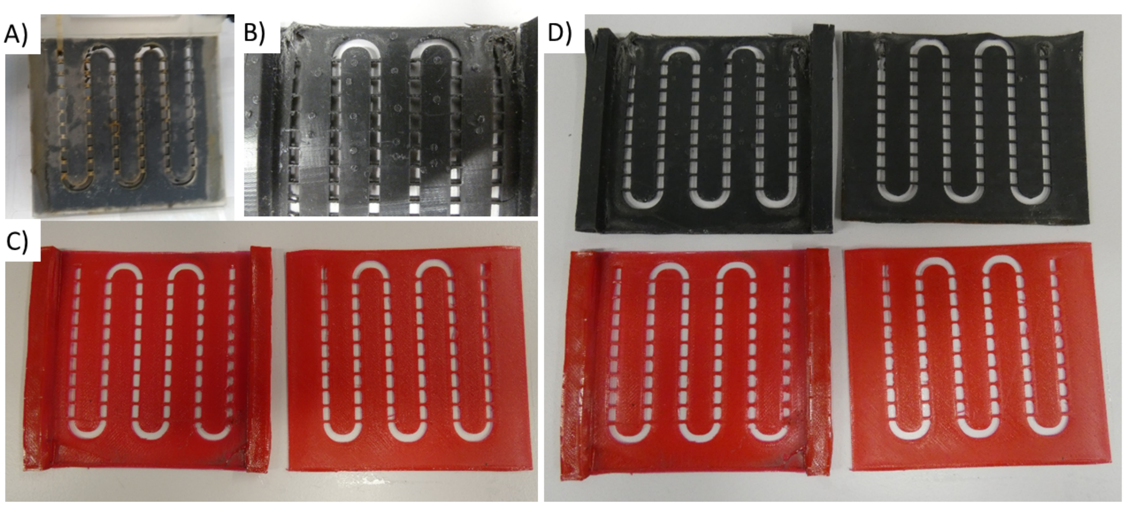

Figure S1 A) Flexible resin gaskets degraded after working without limited current. B) Flexible resin gaskets status after a batch of experiments. C) TPU gaskets after a batch of experiments D) Comparison of the status between flexible resing and TPU gaskets.

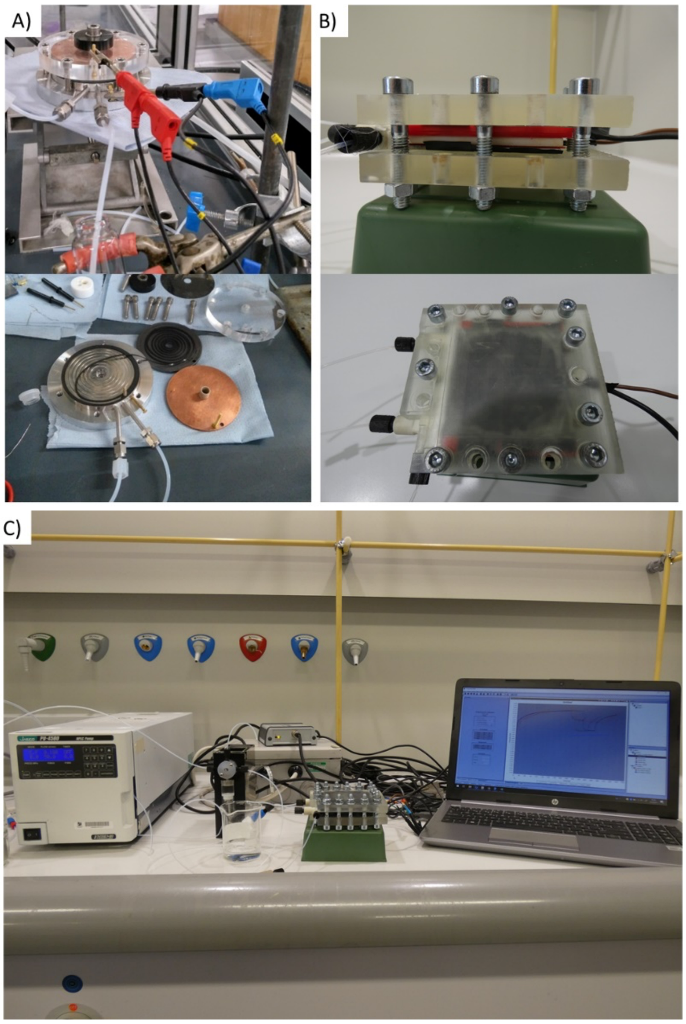

Figure S2 A) Ammonite8 Spiral Reactor B) 3D Printed ECOBR C) Setup used for the ECOBR experiments.

2 Programming of the syringe pump used for oscillation

A Labview VI was developed to control the syringe pump that generates the oscillating flow. The front panel for the executable can be seen at figure S5A, there the user can input the desired oscillating conditions. The block diagram comprises a series of cases that activate different functions, including the start/stop of the oscillation and other processes like pump cleaning or initialization, an example can be seen at figure S5B. Finally, the oscillation order is sent to the pump as a string of commands in the required format generated using the code seen in figure S5C.

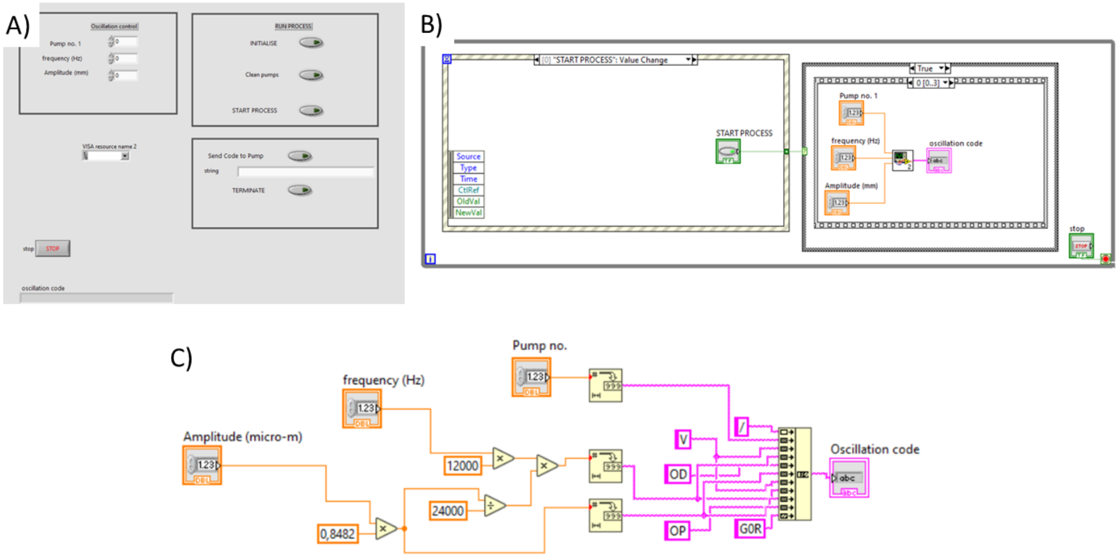

Figure S3 A) Front panel of the VI executable where user can select conditions. B) Part of the block diagram. C) Block diagram for the generation of the oscillation code.

3 CV of the oxidation of Tris-HCl

The voltage variations recorded in the proof of concept experiments observed were presumably due to the oxidation of Tris-HCl. A CV for Tris-HCl was performed.

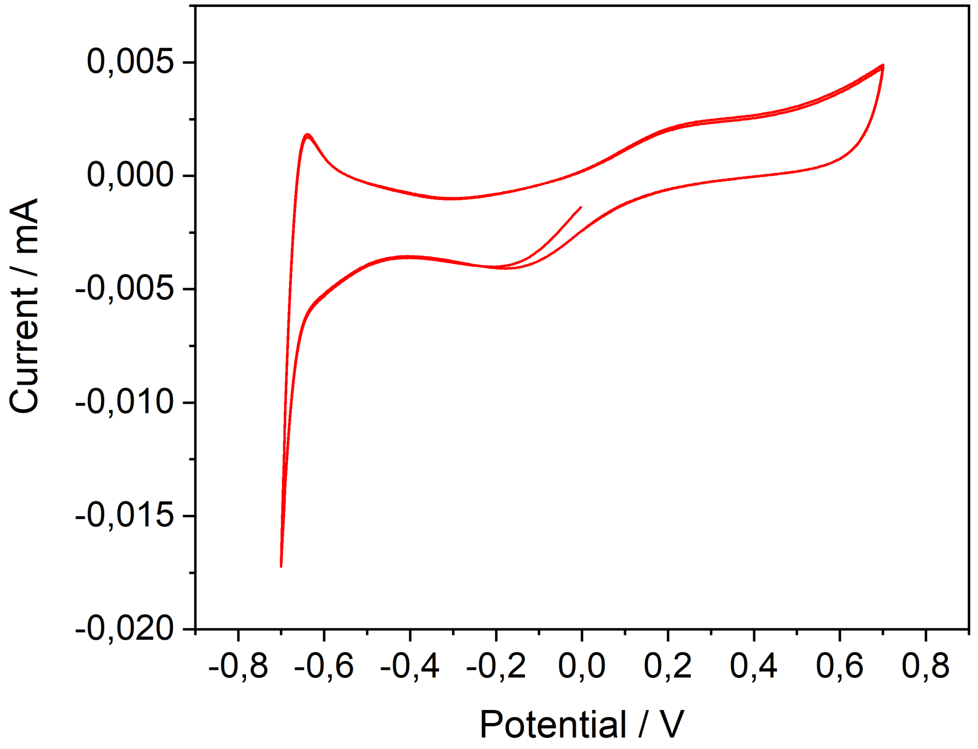

Figure S4 Cyclic voltammetry of the oxidation of NADH

4 Geometry used for the CFD simulations

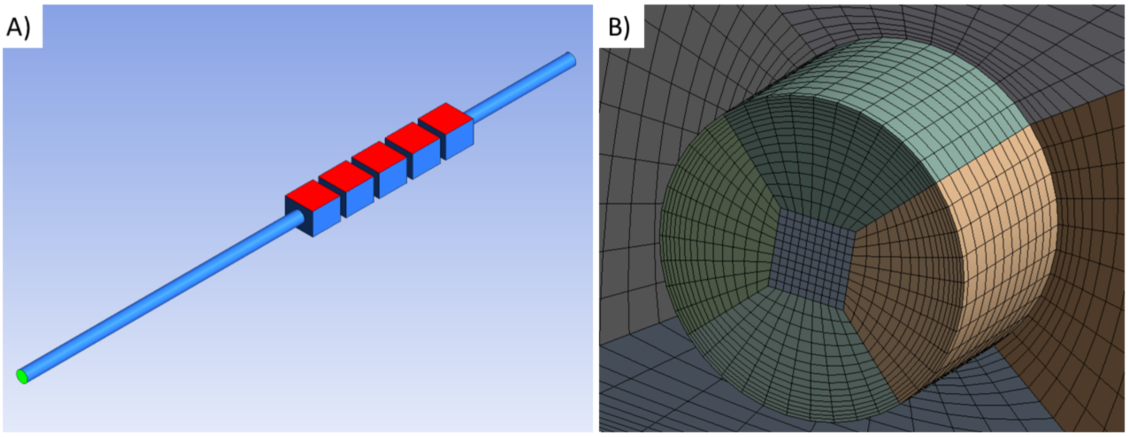

Figure S5 A) Simplified 3D geometry used for the simulation. Red: electrodes. Green: inlet and outlet B) structure of the mesh

To accelerate the calculations, a subset of 5 cells was simulated (figure S4A), with proper inlet and outlet separation to minimize the effects of boundary conditions on the flow inside the cells. The surfaces marked in red represent the cell electrodes. Figure S4B shows a detail of the fully structured 3D mesh that was generated, which is based on the O-GRID approach for the tubular regions.

Simulated flow field for non-oscillatory conditions

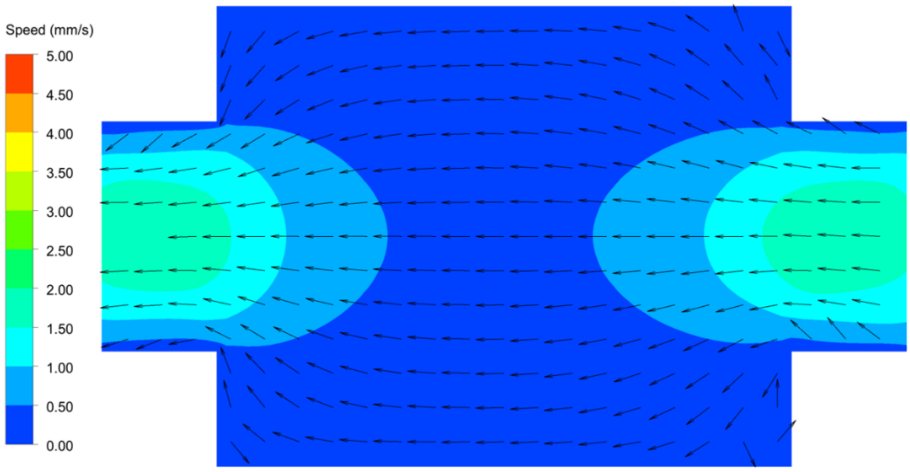

Figure S6 Flow field for the non-oscillatory conditions.

The flow is distributed evenly when the reactor is working under non-oscillatory conditions. Also, flow speed is extremely low and near the electrodes.

5 Study of higher currents in reaction

The use of higher currents lowered the yield presumably due to overoxidation of the substrate. Therefore, definitive experimental currents were selected using Faraday's law, considering the concentration of NADH used, the cell's volume, residence time and flow.

$$i = \frac{m \cdot n \cdot F}{t} \qquad \text{(Eq. S1)}$$

Where i = current, m = mol, n = electron number, F = Faraday constant, t = time.

Table S1 *Results for experiments using higher currents in a commercial Ammonite 8 cell reactor at a concentration of NADH 10 mM.*

**Entry**	**Flow / mL min^{-1}**	**t_{Res} / min**	**I / mA**	**Curr. dens. / mA cm^{-2}**	**Yield / %**
1	0.01	100	0	0.0000	0
2	0.01	100	0.32	0.0160	68.59
3	0.01	100	0.64	0.0320	39.48
4	0.01	100	1.29	0.0645	23.08
5	0.01	100	2.57	0.1285	0

6 NAD+ quantification

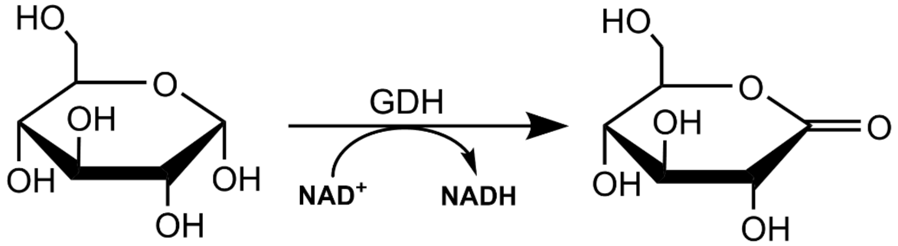

Figure S7 Reaction used for the NAD+ quantification

The NADH cofactor oxidation was quantified through its use in the enzymatic transformation of glucose into glucolactone employing GDH enzyme. After the enzymatic reaction, the samples were analysed in UV-vis spectrophotometer at 340 nm and quantified through a calibration curve of NAD+.

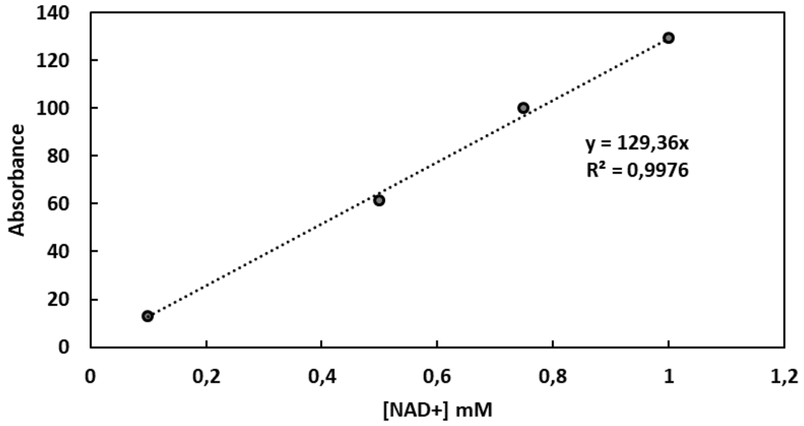

[NAD+] / mM	Absorbance / a.u.
0,1	12,9
0,5	61,4
0,75	99,7
1	129

Figure S8. Calibration curve for NAD+ concentration
